# Two-Year Trajectories of Dental Anxiety in Parents and Their Association with Parents’ and Children’s Oral Healthcare Procedures in FinnBrain Birth Cohort Study

**DOI:** 10.3390/dj12030072

**Published:** 2024-03-07

**Authors:** Satu Lahti, Eeva-Leena Kataja, Auli Suominen, Katri Palo, Mika Ogawa, Anu Kallio, Outi Räikkönen, Vesa Pohjola, Kari Rantavuori, Linnea Karlsson, Hasse Karlsson

**Affiliations:** 1 Department of Community Dentistry, University of Turku, 20014 Turku, Finland; auli.suominen@utu.fi (A.S.); katri.palo@utu.fi (K.P.); mika.ogawa@utu.fi (M.O.); anu.kallio@utu.fi (A.K.); omhagq@utu.fi (O.R.); vesa.pohjola@utu.fi (V.P.); 2 FinnBrain Birth Cohort Study, Department of Clinical Medicine, Turku Brain and Mind Center, University of Turku, 20014 Turku, Finland; eeva-leena.kataja@utu.fi (E.-L.K.); linnea.karlsson@utu.fi (L.K.); hasse.karlsson@utu.fi (H.K.); 3 Centre for Population Health Research, University of Turku, Turku University Hospital, 20521 Turku, Finland; 4 Oral Health Services, Wellbeing Services County of Southwest Finland, 20521 Turku, Finland; 5 Research Unit of Population Health, Faculty of Medicine, University of Oulu, 90014 Oulu, Finland; 6 Department of Oral Development and Orthodontics, University of Turku, 20014 Turku, Finland; kari.rantavuori@utu.fi; 7 Cleft Palate and Craniofacial Center, Department of Plastic Surgery Helsinki University, Helsinki University Hospital, 00014 Helsinki, Finland; 8 Unit of Public Health, Department of Clinical Medicine, University of Turku, 20014 Turku, Finland; 9 Department of Clinical Medicine, Paediatrics and Adolescent Medicine, University of Turku, Turku University Hospital, 20521 Turku, Finland; 10 Department of Psychiatry, University of Turku, Turku University Hospital, 20521 Turku, Finland

**Keywords:** dental anxiety, dental treatment, oral healthcare, treatment procedure, trajectory, mother, father, children

## Abstract

We aimed to identify parents’ dental anxiety trajectories and the association of the trajectories with the number of parents’ and their children’s oral healthcare procedures in the FinnBrain Birth Cohort Study. Dental anxiety was measured with the Modified Dental Anxiety Scale at gestational weeks (gw) 14 and 34, as well as 3 and 24 months (mo) after childbirth. Oral healthcare procedures from gw14 to 24 mo were obtained from the national patient data register and categorized as preventive and treatment. Trajectories were identified with latent growth mixture modelling for 2068 fathers and 3201 mothers. Associations between trajectories and procedures adjusted for education were analyzed using unordered multinomial logit models. Fathers’ trajectories were stable low (80.1%), stable high (3.4%), stable moderate (11.0%), moderate increasing (3.9%) and high decreasing (1.6%). Mothers’ trajectories were stable low (80.7%), stable high (11.2%), moderate increasing (5.3%) and high decreasing (2.8%). Mothers with decreasing dental anxiety had a higher number of preventive and treatment procedures. Fathers with decreasing dental anxiety had a higher number of preventive and treatment procedures, while fathers with increasing dental anxiety had fewer procedures. Children of mothers with stable low dental anxiety had higher number of preventive procedures. There seems to be a two-way association between dental anxiety trajectories and oral healthcare procedures.

## 1. Introduction

Dental anxiety, often used interchangeably with dental fear, consists of different emotional, cognitive, behavioral or physical signs and symptoms related to oral healthcare, and it appears in a continuum from fearlessness to specific phobia [1,2,3]. Dental anxiety can lead to a vicious cycle including the avoidance of oral healthcare, deteriorating oral health and feelings of shame, and it can be transferred from parents to their children, for example, by vicarious learning [1,2,3]. Two components of dental anxiety, anticipatory anxiety and treatment-related anxiety, have been reported, and they seem to capture dental anxiety originating from different sources, referred to as exogenous (external sources such as direct or indirect vicarious experiences) and endogenous (internal sources such as temperament or vulnerability to psychological disorders) [1,4,5].

Dental anxiety is reported by every third adult and has shown stable or decreasing overall trends in adults [6,7,8,9,10]. Interestingly, while the mean levels of adult dental anxiety have shown to decrease at the population level, the prevalence of high dental anxiety has remained fairly stable [7,11]. To our knowledge, to date, the only study that has identified pathways of changes in several time points i.e. trajectories of dental anxiety reports them from age 15 to 32 years. It identified six trajectories [12]. These included stable anxious, stable nonanxious low, stable nonanxious medium, adolescence onset, adult onset and recovery trajectories. The observed trajectories were associated with dental caries experience, childhood caries experience predicting stable high dental anxiety and early adulthood extractions with the adult onset of dental anxiety [12]. Personality traits were also associated with these trajectories. 

Dental anxiety is associated with avoidance or non-habitual dental attendance, as well as poorly perceived and professionally assessed oral health [13,14,15,16]. Parental dental anxiety is associated with child dental anxiety, but they do not seem to change concurrently [9]. Some studies show that those mothers who visit the dentist irregularly tend to bring their children to a dentist irregularly as well [17,18]. Another study reported an association between maternal dental anxiety and their children’s dental service utilization, and the association seemed to be mediated through the mother’s dental utilization [19]. 

Increased dental anxiety led to irregular attendance and vice versa in Finnish adults [20]. In New Zealand, dental anxiety was also a predictor for membership in the opportunist or decliner trajectories of dental attendance [21], which, in turn, were associated with poorer oral health [22]. Changes in the dental anxiety of parents may also affect dental visits and treatment received by their children. However, we could not identify any studies focusing on these associations.

The aims of this study are, first, to identify trajectories of overall dental anxiety and those of its two factors (anticipatory and treatment-related dental anxiety) among parents of the FinnBrain Cohort Study, and secondly, to analyse whether these trajectories are associated with the number of oral healthcare procedures of the parents and their children.

We hypothesize, based on previous findings and clinical experience, that we can identify stable high, stable low, increasing and decreasing dental anxiety trajectories for overall dental anxiety and anticipatory and treatment-related dental anxiety (Hypothesis 1). We also hypothesize that stable high dental anxiety, especially treatment-related dental anxiety, is associated with a higher number of oral healthcare procedures among parents and their children (Hypothesis 2). 

## 2. Materials and Methods

This study is nested in the FinnBrain Birth Cohort Study, which studies genetic and environmental influence on child development and health outcomes. The design is longitudinal. The Intermunicipal Hospital District of Southwest Finland gave ethical clearance for the FinnBrain Cohort Study on 14 June 2011 (14.6.2011 ETMK:57/180/2011 § 168). Adult participants gave signed informed consent for the study for themselves and their children. Participants at the start of the study were recruited among pregnant women and their partners attending ultrasonography appointments in South-Western Hospital District, Finland and Åland Islands, Finland, in 2011–2015. Mothers were asked to invite partners (later called fathers), who did not attend the ultrasonic appointment, to participate in the study. Of those informed about the study (N = 5970), 3808 (66%) mothers and 2623 fathers or other partners of the mother, expecting 3837 children (twins included), agreed to participate. Of those who agreed, 3095 (81%) mothers and 2011 (77%) fathers returned the baseline questionnaire and started the study [23]. 

**Figure 1:**
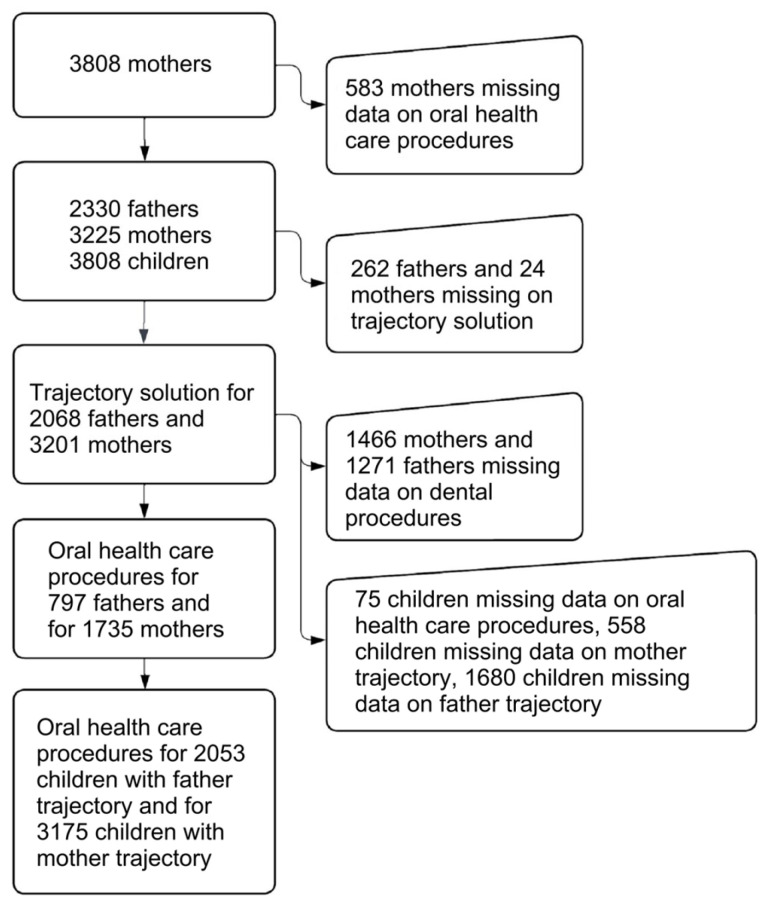
Flowchart of the participants.

The study participants included a subpopulation of mothers and fathers with at least one measurement on dental anxiety and sufficient data on oral healthcare procedures, as well as their children with sufficient data on oral healthcare procedures. The dental anxiety of the parents was measured in four points: during pregnancy, at gestational weeks (gw) gw14 and gw34, and 3 and 24 months (mo) after childbirth. Data about oral healthcare procedures were obtained from the public healthcare centers’ patient data register maintained by The Finnish Institute for Health and Welfare. Data on the oral healthcare procedures of parents and their children were collected between 2013 and 2018 for this study. For children born after 2013, data from birth were used, and for others, from 2013 until 2018. For parents, data on oral healthcare procedures between the gw14 and 24mo data collection points (i.e., between the four dental anxiety measurement points) were used for this study. In the analyses, data for those parents who had at least one dental anxiety measurement or who had oral healthcare procedures during the period were selected. Details of the number of participants included are presented in flowchart in Figure 1.

Dental anxiety was measured with the Finnish version of the Modified Dental Anxiety Scale (MDAS), which has shown good reliability and validity [24,25,26]. The MDAS consists of five items: (1) If you went to your dentist for treatment tomorrow, how would you feel? (2) If you were sitting in the waiting room (waiting for treatment), how would you feel? (3) If you were about to have a tooth drilled, how would you feel? (4) If you were about to have your teeth scaled and polished, how would you feel? (5) If you were about to have a local anaesthetic injection in your gum, above an upper back tooth, how would you feel? The response options range from 1 (not anxious) to 5 (extremely anxious), which are summed to a total score in the range of 5–25, with values 19 and above indicating high dental anxiety [27]. Sums of the two factors established for the MDAS were also calculated: anticipatory dental anxiety (items 1 and 2, score range 2–10) and treatment-related dental anxiety (items 3, 4, and 5, score range 3–15) [4,5].

Oral healthcare procedures were categorized according to the oral health procedure classification which is part of the national healthcare procedure classification maintained by the Institute of Health and Welfare. The categories were examination and treatment plan, preventive oral healthcare, radiological examinations, periodontology, cariology, endodontics, prosthetics, surgery, stomatognathic physiology, orthodontics and anaesthetics. Examination and treatment plan, preventive oral healthcare and radiological examination codes were further categorized as preventive procedures, and filling, periodontal treatment, endodontic treatment and surgery procedure codes were categorized as treatment procedures. Prosthetic, stomatognathic physiology, orthodontic and anaesthetic codes were not included, the first three due to their low number and the latter as it is considered as integral part of oral healthcare treatment in Finland.

The education of the parents was included as a covariate. Data on the parents’ age at childbirth were drawn from the Finnish Medical Birth Register and on education from self-report questionnaires from gw14 [23]. Educational level was categorized as low (high school/vocational), medium (polytechnics) and high (university or comparable).

Trajectory classes were identified separately for mothers and fathers across the four measurement points, as dental anxiety has shown to vary according to gender, especially during pregnancy in this population [28]. Latent growth mixture modelling (LGMM) was conducted in Mplus [29] to identify subpopulations based on their trajectories of dental anxiety and its two dimensions. Missing data were handled by using the FIML (Full Information Maximum Likelihood) estimator. Data analysis was restricted to those study members for whom dental anxiety data were available from at least one assessment point. The number of latent classes was determined by first increasing the number of classes in the analysis until the fit of the model was insufficient. Of the sufficiently fit models, selection for further analysis was made, not only on the fit, but also on previous research, interpretability, clinical relevance and ensuring that the number of participants in each class was sufficient to perform further analyses. This selection was consistent with recommendations and guidelines for identifying classes [30]. The fit indices that were used to select the number of classes retained included the Bayesian Information Criterion (BIC) and Akaike Information Criterion (AIC) (lower values indicating a better model), Entropy (with values closer to 1.0 indicating a higher confidence of classification), posterior probabilities of class membership, and Vuong–Lo–Mendell–Rubin likelihood ratio test (VLMR-LRT) and Bootstrapping Likelihood Ratio Test (BLRT) for k versus k − 1 groups (with *p* values lower than 0.05 suggesting that k + 1 is superior in comparison to k groups) (see references for these indices in, e.g., Ram and Grimm, 2009) [30].

The associations between selected trajectories and parental education were examined using cross-tabulations with the Chi-square test, and the associations between trajectories and parents’ and children’s oral healthcare procedures were examined using descriptive statistics with the Jonckheere–Terpstra test. The associations between oral health are procedures and parental education were examined using descriptive statistics with the Jonckheere–Terpstra test. The associations between trajectories and oral healthcare procedures were evaluated using unordered multinomial logit models. The dependent variable was the trajectory of mothers and fathers. The separate analyses were conducted with preventive and treatment procedures as independent variables. Interactions between independent variables and covariate were modelled, and the model correctness was evaluated with the deviance and Pearson goodness-of-fit statistics. The confounding of education was taken into account. The fathers’ models for anticipatory and treatment dental anxiety trajectories were not adjusted due to insufficient sample size. Statistical significance was considered at *p* values lower than 0.05. Mplus 8.0 software was used in the trajectory analyses [29], and IBM SPSS Statistical Package 29.0 Armonk, NY, USA [31] and SAS 9.4, Cary, NC, USA [32] were used in further analyses. 

## 3. Results

The mean age of the fathers was 32.2 years (SD 5.36), and of the mothers, 30.4 years (SD 4.55). The distribution of the participants according to their dental anxiety, education and oral healthcare procedures for themselves and their children are presented in Table 1. The mean dental anxiety at gw14 was 9.2. (SD = 4.2) for those with information about oral healthcare procedures and 9.1 (SD = 4.0) for those without information about oral healthcare procedures between 2013 and 2018 in fathers (*p* = 0.730, *t*-test) and 10.5 (SD = 4.6) and 10.7 (SD = 4.8) in mothers (*p* = 0.827, *t*-test), respectively. 

The fit indices of the different dental anxiety trajectory solutions for fathers and mothers are presented in Table 2. 

**Figure 2:**
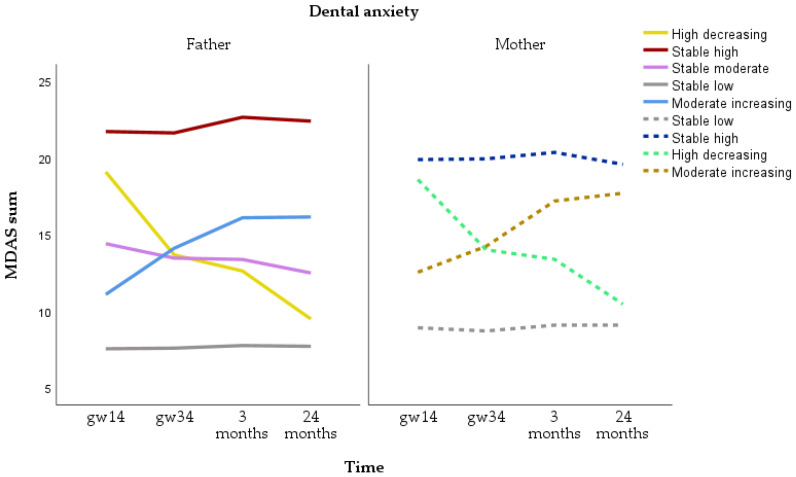
Trajectories of total dental anxiety (sum of the Modified Dental Anxiety Scale) for fathers and mothers.

Trajectories for MDAS sum were identified for n = 2068 fathers and 3201 mothers. For fathers, the five-class model was selected for total dental anxiety. The five categories were stable low (n = 1657, 80.1%), stable high (n = 70, 3.4%), stable moderate (n = 228, 11.0%), moderate increasing (n = 80, 3.9%) and high decreasing (n = 33, 1.6%). For mothers, the four-class model was selected. The four categories were stable low (n = 2583, 80.7%), stable high (n = 360, 11.2%), moderate increasing (n = 169, 5.3%) and high decreasing (n = 89, 2.8%) (Figure 2). The majority of fathers and mother showed stable low dental anxiety. While more mothers than fathers had stable high dental anxiety, the level of anxiety was higher in fathers than in mothers. 

**Figure 3:**
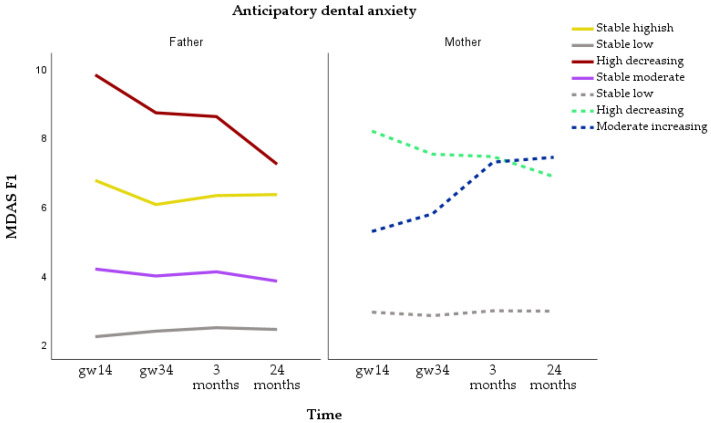
Trajectories of anticipatory dental anxiety (sum of items 1 and 2 of the Modified Dental Anxiety Scale = MDAS F1) for fathers and mothers.

Trajectories for anticipatory dental anxiety were identified for n = 2067 fathers and 3198 mothers. For fathers, the four-class model was selected. The categories were stable low (n = 1355, 65.6%), stable moderate (n = 509, 24.6%), stable highish (n = 158, 7.6%) and high decreasing (n = 45, 2.2%). For mothers, the three-class model was selected. The three categories were stable low (n = 2658, 83.1%), high decreasing (n = 346, 10.8%) and moderate increasing (n = 194, 6.1%) (Figure 3). More mothers than fathers had stable low and high decreasing anticipatory dental anxiety. The increasing anticipatory dental anxiety trajectory was observed only in mothers.

**Figure 4:**
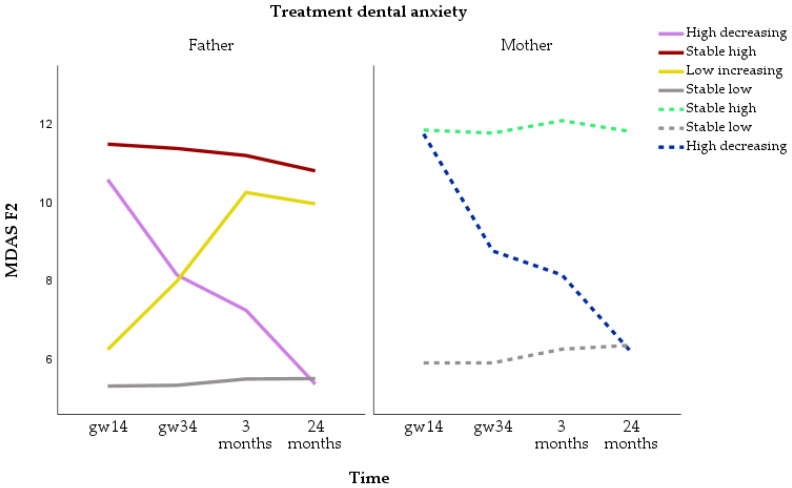
Trajectories of treatment-related dental anxiety (sum of items 3, 4 and 5 of the Modified Dental Anxiety Scale = MDAS F2) for fathers and mothers.

Trajectories for treatment-related dental anxiety were identified for n = 2068 fathers and 3201 mothers. For fathers, the four-class model was selected. The four categories were stable low (n = 1819, 88.0%), stable high (n = 165, 8.0%), low increasing (n = 42, 2.0%) and high decreasing (n = 42, 2.0%). For mothers, the three-class model was selected. The three categories were stable low (n = 2592, 81.0%), stable high (n = 540, 16.9%) and high decreasing (n = 69, 2.2%) (Figure 4). Mothers showed stable high treatment-related dental anxiety twice as often as fathers. On the other hand, the increasing treatment-related dental anxiety trajectory was observed only in fathers. 

Table 3 shows the differences in oral healthcare procedures for parents and their children according to total dental anxiety trajectory groups. In fathers, the highest number of both preventive and treatment procedures for the self were observed among those who belonged to the stable high trajectory, while in mothers, the highest number of both preventive and treatment procedures for the self were observed among those who belonged to the high decreasing trajectory. For oral healthcare procedures for the child, the only statistically significant association was observed for the trajectories of the mothers. The highest number of preventive procedures for children was observed among mothers with stable low anxiety. 

Table 4 and Table 5 show the differences in oral healthcare procedures for parents and their children according to the anticipatory and treatment-related dental anxiety groups, respectively. In anticipatory dental anxiety trajectories, statistically significant associations were observed with preventive and treatment procedures for the self in mothers. The highest number of procedures were observed in mothers belonging to the high decreasing trajectory. For treatment-related dental anxiety, the only statistically significant association was observed among fathers. The highest number of preventive procedures was observed in fathers belonging to the stable highish trajectory. 

The educational level of both parents varied according to the trajectories (Table 6). Fathers with a low level of education were the majority in all trajectories, but especially in the stable high and high decreasing trajectories. In mothers, those with low education were the majority, especially in the stable high and high decreasing trajectories, while in the other two, the distribution of mothers by educational level was more equal. 

The number of oral healthcare procedures varied according to educational level both in fathers and mothers. The mean (and median) numbers of treatment procedures for those having a low, medium and high educational level were 2.78 (2.0), 2.43 (2.0) and 2.03 (1.0) (*p* = 0.013) for fathers and 3.16 (2.0), 2.50 (2.0) and 1.84 (1.0) (*p* < 0.001) for mothers, respectively. For preventive procedures, the mean (and median) numbers for those having a low, medium and high educational level were 3.61 (3.0), 3.17 (2.0) and 2.62 (2.0) with *p* = 0.013 for fathers and 3.80 (3.0), 3.12 (2.0) and 2.66 (2.0) with *p* < 0.001 for mothers, respectively.

When adjusted for education, mothers in the high decreasing group had a higher number of preventive (OR = 1.12, 95%CI 1.04–1.20) and treatment (OR = 1.12, 95%CI 1.01–1.23) procedures than mothers in the stable low anxiety group. Also, mothers in the stable high group had a higher number of treatment (OR = 1.08, 95%CI 1.02–1.53) procedures than mothers in the stable low anxiety group. Fathers in the moderate decreasing group had fewer preventive (OR = 0.77, 95%CI 0.60–0.99) and treatment (OR = 0.74, 95%CI 0.54–0.99) procedures than fathers in the stable low anxiety group. Also, fathers in the stable high group had a higher number of preventive (OR = 1.18, 95%CI 1.06–1.31) procedures than fathers in the stable low anxiety group. 

When adjusted for education, mothers in the high decreasing anticipatory dental anxiety group had a higher number of preventive (OR = 1.08, 95%CI 1.02–1.15) procedures than mothers in the stable low anxiety group. Fathers in the high decreasing anticipatory dental anxiety group had a higher number of preventive procedures (OR = 1.26, 95%CI 1.12–1.42) and fathers in the stable moderate anticipatory dental anxiety group a higher number of treatment procedures (OR = 1.08, 95%CI 1.01–1.56) than fathers in the stable low anxiety group. Mothers in the high decreasing treatment-related dental anxiety group had a higher number of preventive (OR = 1.13, 95%CI 1.04–1.23) and treatment (OR = 1.14, 95%CI 1.03–1.27) procedures than mothers in the stable low anxiety group. Fathers in the low increasing treatment-related dental anxiety group had fewer treatment (OR = 0.53, 95%CI 0.31–0.91) procedures than fathers in the stable low anxiety group.

## 4. Discussion

While the majority of fathers and mothers belonged to the stable low dental anxiety trajectory, the trajectory groups also differed between fathers and mothers, especially for the two dimensions of dental anxiety. For anticipatory dental anxiety, more mothers than fathers belonged to the stable low and high decreasing trajectories, while the increasing anticipatory dental anxiety trajectory was observed only in mothers. For treatment-related dental anxiety, mothers belonged twice as often to the stable high trajectory than fathers, while the increasing trajectory was observed only in fathers. These findings supported Hypothesis 1.

This study reported novel associations between trajectories of dental anxiety and oral healthcare procedures. Contrary to Hypothesis 2, in mothers, decreasing dental anxieties were associated with a higher number of preventive and treatment procedures. In fathers, decreasing anticipatory dental anxiety was associated with a higher number of preventive and treatment procedures, while increasing trajectories were associated with fewer procedures. Children of mothers with stable low total dental anxiety had the highest number of preventive procedures. 

The fit indices allowed choosing different trajectory solutions. The differences between the best and poorest AIC and BIC values were only 1%. Also, entropy values were rather similar. Thus, the VLMR-LT and BLRT indices guided the selection of the number of solutions in addition to previous research, interpretability and clinical relevance. Our findings of four and five trajectories were relatively similar to those on six trajectories reported previously [12], partly due to selection criteria. The differences may also be due to a shorter follow-up period of 2 compared to 17 years, possibly also explaining the differences in percentages of participants in different trajectories. Differences may also be due to differences in the age of participants and study year in the beginning of the study. On the other hand, the majority of adult Finns aged 30 to 100 years (88% of males and 82% of females) had low dental anxiety in an 11-year follow-up period, which is a similar finding to the one in this study [6]. The differences in the trajectories of mothers and fathers might be due to the pregnancy issue, as suggested before [28], or due to their different oral healthcare visiting patterns, with women visiting more often than men [33]. Additionally, the association between generalized anxiety and depressive symptoms and the anticipatory dental anxiety has shown to vary between men and women [5], which may also partly explain the difference in the trajectories.

Of adult Finns in 2011, women, those with regular check-ups or those not reporting perceived treatment need were more likely to visit public health dentists than men and those with irregular check-ups or perceived treatment need [33]. In Finland, parents expecting their first child are entitled by legislation [34] to subsidized public dental care visits, including examinations and preventive and needed care. Expecting parents in this area were also reminded about this possibility during free regular mother and child healthcare visits. This special attention during pregnancy might have increased the motivation and likelihood of men, less regular attenders and those not using oral healthcare services despite perceived need to attend. 

This special attention might partly explain the differences in dental anxiety trajectories and in the associations between dental anxiety trajectories and the number of oral healthcare procedures between mothers and fathers. The highest number of oral healthcare procedures among fathers with the stable high dental anxiety trajectory might be due to the fact that those avoiding care due to dental anxiety were now reached and treated. In mothers, a higher number of oral healthcare procedures was systematically associated with decreasing dental anxiety trajectories, while in fathers, only in those with high decreasing anticipatory dental anxiety. While the difference between mothers and fathers is interesting, the association might be explained by desensitization or finding treatment less frightening. Interestingly, in fathers, fewer oral healthcare procedures were observed in those with moderate increasing total dental anxiety and low increasing treatment-related dental anxiety. This might be due to some fathers not completing treatment due to increasing dental anxiety. In mothers, the increasing and decreasing anticipatory dental anxiety trajectories might reflect changes in general anxiety during pregnancy, as these have shown to change simultaneously in the same cohort [27]. The finding that the number of oral healthcare procedures for children and their parents’ dental anxiety trajectories were not, in general, associated is a positive one, suggesting parental dental anxiety is not reflected in children’s oral health. Somewhat surprisingly, children of mothers with stable low total dental anxiety had the highest numbers of preventive procedures, which also include check-ups. It could be that mothers with low anxiety bring their children more frequently to check-ups. In addition, children at this age have generally not had many treatment procedures, and the associations might show at a later age. 

One strength of this study was a large sample of both parents and their children representing the general population in this area from different socio-economic backgrounds [35]. Another strength is that data have been systematically collected with validated questionnaires such as MDAS [24,25,26]. This study has also limitations. Parents with high education were to some extent overrepresented compared to the Finnish population [23]. Also, we could not obtain oral healthcare data from one third of the adults who reported their dental anxiety. In Finland, all adults are entitled to subsidized and children to free public oral healthcare services, but services are congested. Thus, those adults who are better off are more likely to use private oral healthcare services, which are less subsidized. However, the dental anxiety level was similar in those with public oral health visits and in those without them for fathers and mothers. Some of those without visits to public oral healthcare might have been those with high dental anxiety and who are likely to avoid dental visits [20]. Some might have been those who have used private oral health services and were likely to have a higher education level [33], who, in turn, are less likely to report dental anxiety [6]. During years 2011 to 2019, 11–14% of 20–35-year-olds in this area (South-Western Finland) had used private oral healthcare services [35]. Pregnancy might also have influenced changes in dental anxiety in addition to the treatment received [28]. Thus, these results need to be repeated in other populations at another point of life and preferably with a longer follow-up. 

As the findings indicate that dental anxiety may increase or decrease also during adulthood, oral healthcare personnel should first of all assess dental anxiety with validated measures as suggested by previous research [36,37]. There are also easy methods for handling dental anxiety [38], and of these information about procedures, relaxation techniques and time-structuring could be used as preventive methods as well. Those who were assessed as dentally anxious, especially fathers and those with anticipatory dental anxiety, could be included into regular recalls in addition to treating their dental anxiety. Targeting these actions to parents expecting a baby could also have a broader impact in helping to prevent their child’s dental fear. Ensuring that parents take their children to regular check-ups helps children to get used to oral healthcare. This could further prevent oral diseases like dental caries.

## 5. Conclusions

Dental anxiety may increase or decrease over time. There seems to be a two-way association between dental anxiety trajectories and oral healthcare procedures. More procedures in those with decreasing trajectories suggest that oral healthcare acts as exposure to decrease dental anxiety. For those with increasing trajectories, fewer procedures may indicate disrupting treatment and further avoidance of care. However, more studies are needed to assess the causal relationship and factors related to this association. 

## Figures and Tables

**Table 1:** Distribution of the participants according to their dental anxiety at different time points, age at childbirth and oral healthcare procedures for parents and their children over two years.

	Fathers				Mothers			
	n	Mean	SD	Median	n	Mean	SD	Median
Dental anxiety gw14	1930	9.13	4.07	8	3022	10.63	4.70	9
Dental anxiety gw34	1480	8.98	3.95	8	2584	10.34	4.53	9
Dental anxiety 3 mo	1278	9.24	4.09	8	2225	10.85	4.75	10
Dental anxiety 24 mo	676	8.95	3.84	8	1358	10.65	4.60	10
Anticipatory dental anxiety gw14	1930	3.24	1.74	2	3010	3.63	2.03	3
Anticipatory dental anxiety gw34	1478	3.12	1.62	2	2580	3.47	1.92	3
Anticipatory dental anxiety 3 mo	1276	3,233	1.75	2	2222	3.68	2.06	3
Anticipatory dental anxiety 24 mo	673	3.07	1.65	2	1349	3.56	1.95	3
Treatment-related dental anxiety gw14	1935	5.89	2.60	6	3022	7.00	2.97	6
Treatment-related dental anxiety gw34	1480	5.87	2.61	6	2583	6.87	2.90	6
Treatment-related dental anxiety 3 mo	1280	6.02	2.63	6	2225	7.17	2.99	6
Treatment-related dental anxiety 24 mo	676	5.88	2.50	6	1358	7.10	2.95	6
No of preventive t procedures, own	647	3.01	3.09	2	1473	3.30	3.54	2
No of treatment procedures, own	647	2.86	3.09	2	1473	2.59	2.93	2
No of preventive procedures, for child	2053	3.16	2.95	3	3175	3.35	2.97	3
No of treatment procedures, for child	2053	0.05	0.35	0	3175	0.06	0.36	0
Education at gw14	n	%			n	%		
Low	959	48.9			1150	37.7		
Medium	515	26.2			889	29.1		
High	489	24.9			1015	33.2		

gw = gestational week; mo = month; No = number.

**Table 2:** Fit indices for the different dental anxiety trajectory solutions.

	Fathers					Mothers				
	AIC ^4^	BIC ^5^	Entropy	VLMR-LRT ^6^	BLRT ^7^	AIC	BIC	Entropy	VLMR-LRT	BLRT
MDAS ^1^ sum, 1 trajectory	26,226.00	26,276.70	-	-		45,825.84	45,880.48	-	-	
MDAS sum, 2 trajectories	25,661.50	25,729.12	0.922	<0.0001	<0.0001	44,986.39	45,059.24	0.876	<0.0001	
MDAS sum, 3 trajectories	25,499.26	25,583.77	0.847	0.1013	0.1092	44,759.63	44,850.70	0.830	0.0001	0.0001
MDAS sum, 4 trajectories	25,329.73	25,431.15	0.855	0.1211	0.1283	44,548.87	44,658.15	0.847	0.0006	0.0008
MDAS sum, 5 trajectories	25,217.83	25,336.15	0.852	0.0205	0.0228	44,396.04	44,523.54	0.818	0.0016	0.0020
MDAS sum, 6 trajectories	25,167.30	25,302.52	0.809	0.1274	0.1360	44,302.22	44,371.67	0.828	0.2341	0.2429
MDAS F1 ^2^, 1 trajectory	17,466.87	17,517.58	-	-	-	31,291.77	31,346.41	-	-	-
MDAS F1, 2 trajectories	16,637.24	16,704.85	0.944	<0.0001	<0.0001	30,047.98	30,120.82	0.926	<0.0001	<0.0001
MDAS F1, 3 trajectories	16,334.77	16,419.28	0.862	0.0105	0.0122	29,691.16	29,782.22	0.896	0.0001	0.0001
MDAS F1, 4 trajectories	15,842.06	15,943.47	0.941	0.0010	0.0012	29,233.21	29,342.50	0.884	0.0022	0.0027
MDAS F1, 5 trajectories	15,549.42	15,667.73	0.925	0.0026	0.0032	28,918.82	29,046.29	0.881	0.0081	0.0094
MDAS F1, 6 trajectories	15,384.07	15,519.28	0.918	0.2893	0.3040	28,683.28	28,828.96	0.905	0.0921	0.0998
MDAS F2 ^3^, 1 trajectory	22,049.80	22,100.51	-	-	-	38,576.10	38,630.74	-	-	-
MDAS F2, 2 trajectories	21,676.23	21,743.84	0.877	0.0077	0.0091	38,027.19	38,100.05	0.812	<0.0001	<0.0001
MDAS F2, 3 trajectories	21,588.01	21,672.53	0.882	0.2902	0.2995	37,936.18	38,027.25	0.834	0.0001	0.0001
MDAS F2, 4 trajectories	21,491.42	21,592.84	0.865	0.0003	0.0004	37,786.32	37,895.60	0.773	0.0001	0.0001
MDAS F2, 5 trajectories	21,434.38	21,552.70	0.808	0.0798	0.0882	37,721.11	37,848.61	0.748	0.0454	0.0501
MDAS F2, 6 trajectories	21,410.23	21,545.45	0.827	0.0211	0.0239	37,685.35	37,831.06	0.742	0.1617	0.1702

^1^ MDAS = Modified Dental Anxiety Scale; MDAS sum = total dental anxiety sum; MDAS ^2^ F1 = anticipatory dental anxiety sum; MDAS ^3^ F2 = treatment-related dental anxiety sum; ^4^ AIC = Akaike Information Criterion; ^5^ BIC = Bayesian Information Criterion; ^6^ VLMR-LRT = Vuong–Lo–Mendell–Rubin likelihood ratio test; ^7^ BLRT = Bootstrapping Likelihood Ratio Test.

**Table 3:** Mean and median numbers of preventive and treatment procedures for parents and children according to total dental anxiety trajectories.

Trajectories for Total Dental Anxiety	Preventive Procedures				Treatment Procedures			
	For Parent		For Child		For Parent		For Child	
	Mean	Median	Mean	Median	Mean	Median	Mean	Median
**Fathers**								
Stable low	2.96	2.0	3.15	3.0	2.86	2.0	0.06	0.0
Stable high	5.57	4.0	3.58	4.0	4.09	3.0	0.09	0.0
Stable moderate	2.97	2.0	2.98	3.0	2.84	2.0	0.02	0.0
Moderate increasing	1.48	1.0	3.56	4.0	1.38	1.0	0.01	0.0
High decreasing	3.40	3.0	2.91	2.0	3.33	2.0	0.18	0.0
*p*-value	<0.001		0.248		0.043		0.915	
**Mothers**								
Stable low	3.11	2.0	3.40	3.5	2.43	2.0	0.06	0.0
Stable high	3.83	3.0	3.24	3.0	3.41	2.0	0.06	0.0
Moderate increasing	3.87	3.0	3.07	3.0	2.52	2.0	0.02	0.0
High decreasing	6.30	4.0	2.80	3.0	4.51	3.0	0.09	0.0
*p*-value	<0.001		0.019		<0.001		0.431	

*p*-value for Jonckheere–Terpstra test.

**Table 4:** Mean and median numbers of preventive and treatment procedures for parents and children according to anticipatory dental anxiety trajectories.

Trajectories for Anticipatory Dental Anxiety	Preventive Procedures				Treatment Procedures			
	For Parent		For Child		For Parent		For Child	
	Mean	Median	Mean	Median	Mean	Median	Mean	Median
**Fathers**								
Stable low	2.78	2.0	3.21	3.0	2.68	2.0	0.05	0.0
Stable moderate	3.25	3.0	2.96	3.0	3.08	2.0	0.06	0.0
Stable highish	3.38	3.0	3.29	2.0	3.29	2.0	0.03	0.0
High decreasing	6.40	4.0	4.00	4.0	4.60	5.0	0.22	0.0
*p*-value	0.244		0.228		0.346		0.319	
**Mothers**								
Stable low	3.16	2.0	3.38	3.0	2.46	2.0	0.06	0.0
Moderate increasing	3.72	3.0	3.07	3.0	2.50	2.0	0.03	0.0
High decreasing	4.22	3.0	3.20	3.0	3.66	2.0	0.08	0.0
*p*-value	0.002		0.127		0.001		0.596	

*p*-value for Jonckheere–Terpstra test.

**Table 5:** Mean and median numbers of preventive and treatment procedures for parents and children according to treatment-related dental anxiety trajectories.

Trajectories for Treatment-Related Dental Anxiety	Preventive Procedures				Treatment Procedures			
	For Parent		For Child		For Parent		For Child	
	Mean	Median	Mean	Median	Mean	Median	Mean	Median
**Fathers**								
Stable low	2.92	2.0	3.13	3.0	2.83	2.0	0.05	0.0
Stable High	3.93	3.0	3.53	3.5	3.48	2.0	0.05	0.0
Low increasing	1.85	2.0	3.64	4.0	0.92	1.0	0.02	0.0
High decreasing	3.71	3.0	2.79	2.5	3.35	3.0	0.10	0.0
*p*-value	0.021		0.297		0.795		0.714	
**Mothers**								
Stable low	3.14	2.0	3.39	3.0	2.47	2.0	0.06	0.0
Stable high	3.79	3.0	3.18	3.0	3.00	2.0	0.06	0.0
High decreasing	5.97	4.5	3.38	3.0	4.17	3.5	0.06	0.0
*p*-value	0.351		0.161		0.387		0.404	

*p*-value for Jonckheere–Terpstra test.

**Table 6:** Educational level of participating mothers and fathers by dental anxiety trajectories.

Trajectories for Total Dental Anxiety	Educational Level %		
	Low	Median	High
**Fathers**			
Stable low	47	27	26
Stable high	71	20	9
Stable moderate	47	27	25
Moderate increasing	58	23	18
High decreasing	72	16	12
*p*-value	<0.001		
**Mothers**			
Stable low	34	30	36
Stable high	60	21	19
Moderate increasing	39	31	30
High decreasing	55	25	20
*p*-value	<0.001		

*p*-value for Chi-square test.

## Data Availability

Data not available due to restrictions related to privacy and ethical issues.
